# The Prevalence and Prognosis of Resistant Hypertension in Patients with Heart Failure

**DOI:** 10.1371/journal.pone.0114958

**Published:** 2014-12-09

**Authors:** Chun-Na Jin, Ming Liu, Jing-Ping Sun, Fang Fang, Yong-Na Wen, Cheuk-Man Yu, Alex Pui-Wai Lee

**Affiliations:** Division of Cardiology, Li Ka Shing Institute of Health Sciences, Department of Medicine and Therapeutics, Prince of Wales Hospital, The Chinese University of Hong Kong, Hong Kong SAR, People’s Republic of China; National Cancer Center, Japan

## Abstract

**Background:**

Resistant hypertension is associated with adverse clinical outcome in hypertensive patients. However, the prognostic significance of resistant hypertension in patients with heart failure remains uncertain.

**Methods and Results:**

The 1 year survival and heart failure re-hospitalization rate of 1288 consecutive patients admitted to a university hospital for either newly diagnosed heart failure or an exacerbation of prior chronic heart failure was analyzed. Resistant hypertension was defined as uncontrolled blood pressure (>140/90 mmHg) despite being compliant with an antihypertensive regimen that includes 3 or more drugs (including a diuretic). A total of 176 (13.7%) heart failure patients had resistant hypertension. There was no difference in all cause mortality, cardiovascular mortality, and heart failure related re-hospitalization between patients with versus without resistant hypertension. Diabetes [hazard ratio = 1.62, 95% confidence interval = 1.13–2.34; *P* = 0.010] and serum sodium >139 mmol/L (hazard ratio = 1.54, 95% confidence interval = 1.06–2.23; *P* = 0.024) were independently associated with resistant hypertension. Patients with resistant hypertension had a relatively higher survival rate (86.9% vs. 83.8%), although the difference was not significant (log-rank x^2^ = 1.00, *P* = 0.317). In patients with reduced ejection fraction, heart failure related re-hospitalization was significantly lower in patients with resistant hypertension (45.8% vs. 59.1%, *P* = 0.050).

**Conclusions:**

Resistant hypertension appears to be not associated with adverse clinical outcome in patients with heart failure, in fact may be a protective factor for reduced heart failure related re-hospitalization in patients with reduced ejection fraction.

## Introduction

Hypertension is a major public health problem with a global prevalence ranging from about 20% to 40% [1]–[2]. Resistant hypertension, defined by the USA Joint National Committee (JNC)-7 as failure to achieve goal blood pressure (BP) (>140/90 mmHg for the overall population and >130/80 mmHg for those with diabetes or chronic kidney disease) despite adhering to maximum tolerated doses of three antihypertensive drugs including a diuretics, is associated with a higher risk of cardiovascular morbidity and mortality [3]. A similar definition was adopted by the American Heart Association and the European Society of Cardiology.

Despite a standardized definition, the actual prevalence of resistant hypertension in the general population is difficult to estimate. According to the previous population-based studies [4]–[5], retrospective studies [6]–[7] and outcome trials [8]–[9], resistant hypertension is not uncommon in hypertensive population and the estimated prevalence of resistant hypertension varies from study to study. Moreover, resistant hypertension was associated with a significantly increased risk of adverse cardiovascular events compared with non-resistant hypertension and represents an important public health issue. Heart failure is another ubiquitous cause of mortality and morbidity. There is a significant overlap between patients with heart failure and hypertension. In Enhanced Feedback for Effective Cardiac Treatment (EFFECT) study, where 69% patients with reduced ejection fraction (HFREF) and 31% patients with preserved ejection fraction (HFPEF), nearly 51% of total heart failure patients had evidence of hypertension [10]. The Systolic Hypertension in Elderly Program (SHEP) trial included 4736 people >60 years of age and demonstrated that reducing blood pressure from 170/77 to 143/78 mm Hg reduced heart failure events by 48% [11]. Up to now, there is little data in regard to the prevalence of resistant hypertension in HF patients. When heart failure co-existent with resistant hypertension, the combination is likely to be associated with deleterious consequence.

Accordingly, this study will firstly focus on investigating the prevalence of resistant hypertension and the optimal BP control rate in our heart failure patients both with reduced and preserved ejection fraction (EF). Clinical outcomes, such as 1-year all-cause mortality, cardiovascular mortality and heart failure related re-hospitalization, also will be well assessed in heart failure patients with or without resistant hypertension.

## Methods

### 2.1 Patients population

Consecutive patients presented to a tertiary teaching hospital with either newly diagnosed heart failure or an exacerbation of prior chronic HF were prospectively studied. The diagnosis of heart failure was established according to the clinical Framingham criteria [12]. Patients younger than 18 year-old or refusing to participating in this study were excluded. The study was planned according to the Declaration of Helsinki and approved by Joint Chinese University of Hong Kong - New Territories East Cluster Clinical Research Ethics Committee and all patients provided written informed consent to participate in this study.

### 2.2 Baseline measurements

Demography characteristics and clinical data, including the medical history, medications, cardiovascular risk factors, and associate co-morbidities, were collected using a standardized case report form that was completed at the every study visit. Complementary data collection included electrocardiography, echocardiography, and laboratory tests during the follow up visits. Discharge prescription of the main cardiovascular therapeutics classes was recorded.

Baseline BP was measured in seated position after at least 5 minutes of rest. BP measurement in atrial fibrillation, particularly when the ventricular rhythm is highly irregular, will at best constitute a rough estimate, the validity of which can perhaps be improved upon only by using repeated measurements. The treatment goal of hypertension is to maintain the systolic BP (SBP) and diastolic BP (DBP) below 140 mmHg and 90 mm Hg, respectively. Antihypertensive medications were grouped based on drug class, mainly including diuretics, angiotensin-converting enzyme inhibitors (ACEIs)/angiotensin II receptors blockers (ARBs), calcium channel blockers (CCBs), beta-blockers and aldosterone receptor antagonists(ARAs). Resistant hypertension was diagnosed as uncontrolled BP despite using of more than 3 antihypertensive medications, including a diuretics.

Comprehensive two-dimensional Doppler transthoracic echocardiography was performed in all participants (Vivid Five or Seven, General Electric, Milwaukee, WI, USA) using a 2.5 MHz probe. All images were digitally stored with at least three cardiac cycles for off-line analysis. The left ventricular (LV) volumes and left ventricular ejection fraction (LVEF) were assessed by the biplane Simpson’s method. At least three consecutive beats in sinus rhythm were measured and averaged. Patients with an EF of <50% were classified as HFREF, and those with an EF of greater than or equal to 50% were classified as HFPEF.

### 2.3 Ascertainment of outcomes

The primary outcome for the study was all-cause mortality within 1-year follow-up. Secondary outcomes included cardiovascular death and heart failure related re-hospitalization within 1-year follow-up. Outcomes were not adjudicated, but based on physician reporting at the time of follow-up. Cardiovascular death included fatal stroke, fatal myocardial infarction, death attributed to congestive heart failure, sudden cardiac death, pulmonary embolism and other cardiac death.

### 2.4 Statistical analysis

Continuous variables are expressed as the mean ± SD, categorical data are presented as the absolute numbers and percentages. Differences between groups were evaluated using the Student t-test for continuous data, the chi-square test for binomial or nominal variables (or Fisher’s exact test, if appropriate). Logistic regression was conducted to screen factors at baseline associated with resistant hypertension. Hazard ratios (HR) and their 95% confidence interval (CI) were calculated. Kaplan-Meier survival curves were constructed to demonstrate 1-year survival in patients with or without resistant hypertension. The log-rank test was used to determine if actuarial survival was significantly different. Statistical significance was considered as a 2-tailed probability of <0.05. Statistical analysis was performed using SPSS software, version 20.0 for Windows (SPSS, Inc., Chicago, IL, USA).

## Results

### 3.1 Baseline characteristics and follow-up endpoints of the sample

1288 patients with heart failure enrolled in this registry (May 2006 to December 2010) were entered into the final analysis. Among all the patients included, mean age was 75.2±11.9 years, ranged from 31–102 years; 713 (55.4%) patients were female; 519 (40.3%) were HFREF patients; 381 (29.6%) patients received more than 3 antihypertensive medications; 679 (52.7%) patients reached optimal BP target; a total of 176 (13.7%) heart failure patients were found to combine with resistant hypertension. All patients were followed up for 1 year, 203 (15.8%) patients died within 1-year follow-up, among them 45.3% (92) patients were belong to cardiovascular death; 657 (51.0%) patients had at least 1 episode of heart failure related re-hospitalization.

### 3.2 Differential characteristics and the prognosis of patient groups defined by with or without resistant hypertension

Among all the heart failure patients with resistant hypertension, the 5 most used classes of antihypertensive agents included diuretics (100%), beta-blockers (86.4%), ACEIs/ARBs (84.1%), CCBs (31.2%), and ARAs (13.1%). The baseline characteristics for patients with or without resistant hypertension were shown in **Table 1**. Patients with resistant hypertension had a higher incidence of diabetes, a remarkably higher level of serum sodium and albumin, and were more commonly prescribed with statins. Logistic-regression analysis further revealed that patients with higher serum sodium (>139 mmol/L) (HR = 1.54, 95% CI = 1.06–2.23; *P* = 0.024) and with a history of diabetes (HR = 1.62, 95% CI = 1.13–2.34; *P* = 0.010) at baseline were more likely to be resistant hypertension (**Table 2**).

**Table 1:** Baseline characteristics for heart failure patients with or without resistant hypertension.

Parameters	Non-RHTN(n = 1112)	RHTN(n = 176)	*P*
Age, years	75.3±11.9	74.4±11.8	0.338
Female, no. (%)	612(55.0)	101(57.4)	0.560
NYHA III-IV, no. (%)	799(77.7)	131(80.4)	0.448
Smokers, no. (%)	348(32.1)	48(28.7)	0.381
Heart rate, bpm	90±24	91±24	0.862
LVEF, %	51±16	52±15	0.308
HF with preserved EF, no. (%)	642(58.4)	112(65.1)	0.096
SBP, mmHg	134±25	157±18	<0.001
DBP, mmHg	72±14	81±15	<0.001
**Laboratory Test**			
Sodium, mmol/L	138.9±4.5	139.8±3.6	0.007
Potassium, mmol/L	4.07±0.62	4.05±0.55	0.602
Creatinine, umol/L	138.5±96.7	145.0±116.7	0.424
Blood urea nitrogen, mmol/L	10.1±6.2	9.8±5.8	0.551
Albumin (>34 g/L), no. (%)	827(76.6)	137(83.0)	0.067
**Medical history, no. (%)**			
New on-set HF	644(57.9)	102(58.0)	0.992
Hypertension	826(74.3)	145(82.4)	0.020
Diabetes	414(37.3)	93(52.8)	<0.001
Coronary heart disease	341(29.6)	54(30.7)	0.779
Atrial fibrillation	319(28.9)	43(24.4)	0.220
COPD/Asthma	123(12.5)	11(7.5)	0.081
Stoke/TIA	157(14.2)	28(15.9)	0.538
Hyperlipidemia	194(17.6)	35(20.1)	0.413
Chronic Kidney Disease	182(16.5)	31(17.6)	0.705
Anemia	450(41.1)	63(37.3)	0.342
**Medication, no. (%)**			
Regular nitrates	252(22.8)	42(24.4)	0.645
Digoxin	164(14.8)	31(18.0)	0.278
Aspirin	596(53.6)	99(56.6)	0.470
Wafarin	181(16.4)	30(17.4)	0.727
Statins	369(33.2)	73(41.7)	0.028

NYHA indicates New York Heart Association; LVEF, left ventricular ejection fraction; HF, heart failure; SBP, systolic blood pressure; DBP, diastolic blood pressure; RHTN, resistant hypertension; COPD, chronic obstructive pulmonary disease; and TIA, transient ischemic attack.

**Table 2:** Logistic-regression of resistant hypertension for heart failure patients.

Variable	Univariate analysis			Multivariate analysis		
	HR	95%CI	*P*	HR	95%CI	*P*
Albumin(>34 g/L)	1.49	0.97–2.29	0.069	-	-	-
Sodium(>139.5 mmol/L)	1.50	1.08–2.09	0.016	1.54	1.06–2.23	0.024
Statins used	1.44	1.04–1.99	0.029	-	-	-
Diabetes	1.88	1.37–2.59	<0.001	1.62	1.13–2.34	0.010
COPD/Asthma	0.57	0.30–1.08	0.085	-	-	-

HR indicates hazard ratio; CI, confidence interval; and COPD, chronic obstructive pulmonary disease.

Kaplan-Meier analysis showed that patients with resistant hypertension had a relatively higher survival rate (86.9% vs. 83.8%), while the difference was not significant (log-rank x^2^ = 1.00; *P* = 0.317) at 1-year follow-up. There were no difference in heart failure related re-hospitalization (51.4% vs. 48.3%; *P* = 0.438) and cardiovascular mortality (43.9% vs. 56.5%; *P* = 0.252) between the two groups during a follow-up of 1 year (**Table 3**).

**Table 3:** 1-year outcomes comparison for heart failure patients with or without resistant hypertension.

Outcomes, no. (%)	Heart failure patients		*P*
	Non-RHTN (n = 1112)	RHTN (n = 178)	
All-cause mortality	180(16.2)	23(13.1)	0.291
Cardiovascular mortality	79(43.9)	13(56.5)	0.252
HF Re-hospitalization	572(51.4)	85(48.3)	0.438

HF indicates heart failure; and RHTN, resistant hypertension.

### 3.3 Differential characteristics and the prognosis of patient groups defined by HEREF or HFPEF

In patients with HFREF, the prevalence of resistant hypertension was significantly higher (15.2% vs. 11.4%, *P* = 0.049). Within 1-year follow-up, all-cause mortality was 19.7% in patients with HFREF, significantly higher than patients with HFPEF (19.7 vs. 13.1%, *P* = 0.002); heart failure related re-hospitalization was also higher in patients with HFREF (57.6% vs. 46.6%; *P*<0.001); cardiovascular mortality was similar in both subgroups (49.0% vs. 41.6%; *P* = 0.287). When resistant hypertension was considered, all-cause mortality and cardiovascular mortality were similar in the two groups regardless of patient with reduced or preserved EF. In HFREF group, heart failure related re-hospitalization was significantly higher in patients without resistant hypertension than those with resistant hypertension (59.1% vs. 45.8%; *P* = 0.050), while this difference was not found in HFPEF group (45.9% vs. 50.0%; *P* = 0.415) **(** **Table 4**).

**Table 4:** 1-year outcomes comparison for subgroup analysis based on ejection fraction in patients with or without resistant hypertension.

Outcomes, no. (%)	HFREF patients (n = 519)			HFPEF patients (n = 769)		
	Non-RHTN (n = 460)	RHTN (n = 59)	*P*	Non-RHTN (n = 652)	RHTN (n = 117)	*P*
All-cause mortality	93(20.2)	9(15.3)	0.366	87(13.3)	14(12.0)	0.685
Cardiovascular mortality	46(49.5)	4(44.4)	0.774	33(37.9)	9(64.3)	0.063
HF Re-hospitalization	272(59.1)	27(45.8)	0.050	300(46.0)	58(49.6)	0.477

HFREF indicates heart failure with reduced ejection fraction; HFPEF, heart failure with preserved ejection fraction; and RHTN, resistant hypertension.

## Discussion

### 4.1 The prevalence of resistant hypertension in patients with heart failure

We totally studied 1288 patients with heart failure in the final analysis. The percentage of resistant hypertension in heart failure patients was 13.7%. Compared to patients with HFREF, the prevalence of resistant hypertension was significantly higher in patients with HFPEF. The optimal BP control rate was only 52.7% among all the patients. Patients with a history of diabetes and higher serum sodium (>139 mmol/L) at baseline were more likely to combine with resistant hypertension, which had been well demonstrated as risk factors contributing to resistant hypertension in general population previously [3].

Current prevalence estimated for resistant hypertension in other population subgroups vary. Population-based studies, such as National Health and Nutrition Examination Survey (NHANES), reported that the prevalence of resistant hypertension was 8–12% among adult hypertensive patients (6–9 million people) [13]. Several recent studies estimated the prevalence of resistant hypertension was between 12.8% and 28% among hypertensive patients receiving anti-hypertension treatment [4]–[5], [14]. Hitherto, the prevalence of resistant hypertension has not been well defined in heart failure patients. A greater understanding of the prevalence of resistant hypertension is important to improve the management of these patients. Swedish Heart Failure Registry (S-HFR) had shown that the percentage of heart failure patients with SBP more than 140 mm Hg was 22.4% [15], suggesting that heart failure patients without optimal BP control is common. Our current study firstly reported the overall prevalence of resistant hypertension was 13.7% in heart failure patients, which felled in the range of reported prevalence in hypertensive patients [4]–[5], [14], however this study was not primarily designed as a survey to detect true prevalence rates.

### 4.2 The prognosis of heart failure patients with resistant hypertension within 1-year follow-up

In the current study, heart failure patients with resistant hypertension didn’t show a remarkably difference in the outcomes compared with those without resistant hypertension within 1 year follow up, suggesting that resistant hypertension appears not to be associated with increased 1 year mortality in heart failure patients. In contrast, precious studies had reported that resistant hypertension was associated with a significantly increased risk of adverse cardiovascular events in the rest of cardiac population [6]. Patients present with a long-standing history of poorly controlled hypertension had an unfavorable prognosis. When considering the different study population between ours and others, it is difficult to establish the exact role resistant hypertension had taken in heart failure patients.

Our study had shown the status of resistant hypertension, in another word, elevation of blood pressure above normal in heart failure patients, appears not to be associated with increased 1-year mortality in heart failure patients. As far as we are aware, the result we found was in keeping with the previous studies on heart failure patients. On one hand, a recent meta-analysis quantifying the paradoxical effect of higher SBP on mortality in chronic heart failure (mainly including HFREF) disclosed that the decrease in mortality rates associated with a 10 mm Hg higher SBP was 13.0% (95% CI: 10.6% to 15.4%) in the heart failure population [16]. On the other hand, in several clinical trials involving patients with chronic heart failure [17] or acute heart failure [18]–[19], lower BPs were generally recognized to be an adverse prognostic marker in risk assessment of heart failure, which also were confirmed in both HFREF [20] and HFPEF [21]. Up to now, there was no clear cutoff value to define the optimal SBP control goal in heart failure patients. Data from the African-American Heart Failure Trial (A-HeFT) indicated best outcomes for the group with baseline SBP 126–140 mm Hg, whereas SBP below the median induced a small but significant increase in mortality, regardless of treatment assignment [22].

Patients with HFREF and HFPEF could potentially be viewed as 2 unique populations. Despite our incomplete understanding of the pathophysiologic progression leading to the development into systolic dysfunction or diastolic dysfunction, the benefits of treating hypertension are clear, and the dramatic improvement in outcomes had been seen when hypertension was controlled [23]. Current study disclosed that in patients with HFREF, all-cause mortality and heart failure related re-hospitalization were significantly higher than patients with HFPEF within 1-year follow-up. Large attention on heart failure patients with or without resistant hypertension had been taken in this study. Within 1-year follow-up, HF related re-hospitalization was lower in HFREF patients with resistant hypertension, which imply that resistant hypertension may not be a factor for increasing re-hospitalization in heart failure patients with reduced EF. Our study further found effects of resistant hypertension on 1-year mortality don’t vary from HFPEF patients to HFREF patients, indicating that resistant hypertension appears not associated with adverse long-term clinical mortality.

### 4.3 Clinical significance

Current study has shown 13.7% heart failure patients accompanying with drug-related resistant hypertension. Compared with heart failure patients without resistant hypertension, those with resistant hypertension received more aggressive antihypertensive treatment but similar outcomes. Resistant hypertension has the similar pathophysiologic mechanism to heart failure, as the excessive activation of renin-angiotensin-aldosterone system and sympathetic nervous system. It was known that heart failure patients with preserved EF benefited limitedly from the current available treatments, such as pharmacotherapy and cardiac resynchronization therapy. Over the past few years, there has been significant interest in device-based therapies, which modify sympathetic nerves activity for the management of patients with resistant hypertension, including renal denervation via radiofrequency catheter ablation of renal sympathetic nerves (RDT) [24] and baroreceptor activation therapy (BAT) [25]–[26]. There may be a theoretical concern in practice that patients with heart failure typically have normal or low BP and may be relying upon contributions from their sympathetic nerves activity to sustain BP and organ perfusion, while RDT and BAT therapy might further lower the BP. So, heart failure patients with resistant hypertension may be the optimal indication for these novel device-bases therapies. If confirmed effective, about one in sixth heart failure patients will benefit from these new therapies in our study.

### 4.4 Limitations

Certain limitations should be considered in the interpretation of the study results. Firstly, the present study used office-based BP measurements alone; ambulatory BP measurements may provide more accurate estimates of resistant hypertension and have been shown to be more prognostic [4]. However, office-based BP measurements reflect current practice and are used routinely in the management of heart failure patients. Another limitation is lack of information on medication dosing since optimal dosing constitutes an integral part of the definition of resistant hypertension, whereas some earlier studies have used a definition identical to ours [4]–[5], [14], [27]. Maximally tolerated/adequate doses was referred by guidelines, but vary from person to person [3]. Besides that, BP is intrinsically related to the heart’s ability to pump and decreases as a result of reduced cardiac output due to heart failure deterioration, which will underestimate the true percentage of resistant hypertension in heart failure patients. Furthermore, the sample size in our study is relatively small and the power to discriminate the difference of 1 year outcomes between patients with or without resistant hypertension is low. The present study population was drawn from a single center, so our finding may not be generalizable to other healthcare system. Finally, another limitation in our study is absence of the adjustment of the presence of moderate and severe diastolic dysfunction and left ventricular hypertrophy (LVH), which have been demonstrated as independently predictors of adverse clinical outcomes in patients with heart failure in the CHARM study [28]–[29]. However, most of the previous studies with the purpose of investigating the outcomes in patients with heart failure did not adjust these two parameters [10], [30]–[41]. Only few studies adjusted them separately, but no positive association was found [42]–[44].

## Conclusions

As far as we know, this is the first study focusing on the prevalence and prognosis of resistant hypertension in heart failure patients. Resistant hypertension is a common finding and appears not to be associated with increased 1-year mortality and heart failure related re-hospitalization in heart failure patients. Further studies are necessary to determine the correct pharmacologic treatment, as well as the effectiveness of some novel device-bases therapies, such as RDT and BAT, for heart failure patients with resistant hypertension.
